# Max-mixed EWMA control chart for joint monitoring of mean and variance: an application to yogurt packing process

**DOI:** 10.1038/s41598-024-61132-0

**Published:** 2024-05-06

**Authors:** Seher Malik, Muhammad Hanif, Muhammad Noor-ul-Amin, Imad Khan, Bakhtiyar Ahmad, Abdelgalal O. I. Abaker, Jumanah Ahmed Darwish

**Affiliations:** 1 National College of Business Administration and Economics, Lahore, Pakistan; 2 COMSATS University Islamabad, Lahore Campus, Lahore, Pakistan; 3 Abdul Wali Khan University Mardan, Mardan, Pakistan; 4 Higher Education Department Afghanistan, Kabul, Afghanistan; 5 Applied College, Khamis Mushait, King Khalid University, Abha, Saudi Arabia; 6 Department of Mathematics and Statistics, College of Science, University of Jeddah, Jeddah, Saudi Arabia

**Keywords:** EWMA, Max mixed EWMA statistics, Max-EWMA control chart, Joint monitoring control chart, Applied mathematics, Pure mathematics, Scientific data, Statistics

## Abstract

The Max-Mixed EWMA Exponentially Weighted Moving Average (MM EWMA) control chart is a statistical process control technique used for joint monitoring of the mean and variance of a process. This control chart is designed to detect small and moderate shifts in the mean and variance of a process by comparing the maximum of two statistics, one based on the mean and the other on the variance. In this paper, we propose a new MM EWMA control chart. The proposed chart is compared with existing control charts using simulation studies, and the results show that the chart performs better in detecting small and moderate shifts in both the mean and variance. The proposed chart can be helpful in quality control applications, where joint monitoring of mean and variance is necessary to ensure a product’s or process’s quality. The real-life application of the proposed control chart on yogurt packing in a cup data set shows the outperformance of the MM EWMA control chart. Both simulations & real-life application results demonstrate the better performance of the proposed chart in detecting smaller shifts during the production process.

## Introduction

Monitoring a process mean and variance is crucial for statistical process control to ensure product quality and process stability. To monitor the mean and variation of a process independently, conventional control charts like Shewhart charts and CUSUM (Cumulative Sum) charts are widely applied. These control charts may be unable to identify the issue if a process mean and variance are shifting. As a result, it is essential to monitor the mean and variance jointly. A variant of the standard exponentially weighted moving average (EWMA) charts used in statistical process control (SPC), modified EWMA control charts are also called adaptive EWMA control charts. Some recent variants of memory type control charts are introduced by^1,2^, and^3^. The EWMA control chart modification aims to increase its sensitivity to detect minute changes while keeping false alarms under control. Observing the exponentially weighted moving average of the process measurements over time, the EWMA control chart maintains track of the process mean. Recent observations are given more weight, making the graphic more responsive to changes in the process mean. The MM EWMA control chart has been suggested as a valuable method for joint monitoring of a process's mean and variance to overcome this problem. The MM EWMA control chart is based on the exponentially weighted moving average (EWMA) method, which calculates a weighted average of historical observations by assigning more weight to current observations. The MM EWMA control chart has become quite popular recently as an effective tool for simultaneous monitoring of process means and variance. Much research has been done on this topic, according to a literature review, which emphasizes the efficiency of this chart to identify small and moderate changes in both the mean and variance. The reverse moving average (RMA) chart was introduced as a new forecasting scheme by^4^, who compared it to other charts when operating under ARMA(1,1), AR(1), and MA(1) processes. When the size of the shift is known in advance^5^, demonstrated the benefit of using weighted moving averages (WMA) for early detection of deviations from the goal. Khoo^6^ proposed the use of the MA chart as a means of monitoring the quantity of non-conforming products. Khoo and Wong^7^ introduced a double MA chart that utilizes double moving averages and proved its superiority compared to the traditional MA chart based on average run lengths. Lin and Chou^8^ developed an economic control chart that utilizes moving averages for observations that are associated with each other. Maghsoodloo and Barnes^9^ examined the MA control chart and analyzed its conditional average run length. Further information regarding MA control charts can be found in references^10–13^. Areepong^14^ studied explicated formulas for the ARL on an MA control chart for monitoring the number of defective products and showed that they were easy to use, calculate, and program. Areepong and Sukparungsee^15^ analyzed closed-form formulas for the ARL on an MA control chart for nonconforming ZIP and showed that it performs better as the value of the spam (w) decreases. Chananet et al^16^. studied an approximate formula for the ARL on an MA control chart with zero-inflated negative binomial data and showed that the new formula is simple and easy to implement. Recently Rakitzis et al^17^. studied ZIPINAR (1) processes via the CUSUM control chart which can track mean shift changes in manufacturing processes. They also discussed the influence of zero-inflated data on the control chart. Later, Areepong^18^ studied explicit expressions for the ARL of an MA control chart for a ZIPINAR(1) model and showed that the performance of the MA control chart is better than the EWMA control chart. Khoo and Wong^7^ introduced a twofold moving average (DMA) control chart that enhances the capability of the MA control chart to detect small to moderate shifts in a process that follows a normal distribution. Alevizakos et al^19^. presented the accurate variance of the double moving average control chart, which was first devised by Khoo and Wong^7^, for the supervision of quality attributes that follow a normal distribution. Sukparungsee et al^20^. recently introduced a combined EWMA-MA control chart, following the approach of Khan et al^21^., for monitoring the location of a process. Raza et al^22^. proposed the variance version of the mix EWMA-MA chart statistic is obtained by taking into account the interdependence among the consecutive moving averages. The control limits of the mixed exponentially weighted moving average (EWMA) and moving average (MA) control chart are updated, and the run-length profile is analyzed through the utilization of Monte Carlo simulations. The results unambiguously demonstrate that the EWMA-MA control chart outperforms alternative control charts in detecting mean shifts, hence providing further validation of the simulated results.

The variable sampling interval (VSI) MA chart outperforms the fixed sampling interval (FSI) MA chart in terms of loss cost, according to^23^, thorough development of an economical design for the VSI MA chart. Khoo and Wong^7^ proposed a double moving average (DMA) control chart that outperforms the MA chart in detecting small to moderate shifts. To identify shifts in the process mean underlying asymmetries and symmetries^20^, introduced Mixed Moving Average-Exponentially Weighted Moving Average Control Charts. The numerical results compared MA-EWMA to other control charts including Shewhart, EWMA, and MA, and discovered that it outperformed them all. EWMA-MA charts were recommended by^20^. The results showed that when comparing the efficiency of the proposed chart to the Shewhart, MA, and EWMA charts, the suggested chart performed better when employing average run length, standard deviation of run length, and median run length for detection. The triple moving average control chart was suggested by^11^ to improve the MA chart's recognition ability. It is shown that the proposed chart surpasses the MA and DMA charts in detecting small to moderate shifts for a small value of span w. When comparing the performance of various charts^24^, proposed a new nonparametric exponentially weighted moving average chart with a progressive setup based on sign and arcsine test statistics, and the research showed that the proposed chart has a much better ability to detect small and persistent shifts. To monitor changes in the process mean under the assumption of normal distribution^25^, explored deeper into the MEWMA chart by modifying the value of its additional design parameter and developed the double MEWMA (DMEWMA) chart. The standard deviation and mean run length were used to evaluate the chart's efficacy. Better detection capacity for the range of shifts was seen in the modified schemes when the smoothing parameter was configured to a medium or large value and the extra parameter was assigned to a negative value, compared to the classical chart^26^. suggests an updated HWMA chart that takes advantage of the cumulative sum chart's underlying statistical plotting model. It is clear from this comparison that the proposed chart outperforms the current alternatives. The Modified Exponentially Weighted Moving Average—Moving Average Control Chart (MMEM) was proposed by^12^ to monitor changes in the process mean. The proposed chart outperformed alternative control charts through both simulated and practical examination. For the (ZIPINAR(1)) model^13^, suggested a DMA double moving average control chart. It was more effective than other control charts at identifying statistically significant changes in the mean. It was suggested by^12^ that a novel nonparametric Tukey modified exponentially weighted moving average—Moving average control chart will improve performance. The proposed chart's standout feature is its superior performance in identifying mild changes.

The literature research demonstrates that the MMEWMA control chart is an effective method for simultaneous monitoring of a process's mean and variation. To ensure the quality of goods and services, the proposed chart has various applications in numerous industries, including manufacturing, healthcare, and finance.

The Max-Mixed EWMA Control Chart is designed to maximize sensitivity to shifts in both the mean and variance of a process. Traditional control charts frequently focus on either mean or variance control individually. However, in many production processes, changes in mean and variance may occur simultaneously. The MM EWMA Control Chart enables the simultaneous monitoring of both parameters, offering a more comprehensive view of the process dynamics. The MM EWMA Control Chart is meant to limit the risk of false alarms, which is critical in industrial processes where excessive alerts can lead to unneeded interventions and disruptions. By combining information on mean and variance in a statistically sound manner, the MM EWMA chart can provide a more accurate estimate of process stability. Simultaneously monitoring both the mean and variation can yield additional information for decision-making. Early detection of changes in both the average and variability enables proactive optimization of the process. To optimize efficiency and enhance output, it is crucial to swiftly resolve any difficulties that arise throughout the yogurt packaging process, allowing for fine-tuning and eliminating wastage. The proposal of the MM EWMA Control Chart for simultaneous monitoring of the mean and variance in a yogurt packaging process can be justified by the aim to promote process comprehension, boost quality control, and facilitate prompt corrective measures to uphold ideal production circumstances.

The remaining article is organized as follows: we explain the existing Max EWMA control chart in section "Max-EWMA control chart for joint monitoring", and we describe the suggested MM EEWMA control chart for concurrent monitoring in section "Proposed MM EWMA Control Chart for Joint Monitoring". In section "Simulation Study", a simulation study is described to assess the performance of the suggested chart. Section "Illustration example" presents a real-world example of how the suggested chart might be used in practice. Section "Main findings" presents the key findings, and in section "Conclusion", the conclusion is provided.

## Max-EWMA control chart for joint monitoring

The current Shewhart control chart effectively utilizes all the relevant information in the current sample. The EWMA control chart was designed to assign the highest weights to the most recent subgroup, while gradually decreasing the weights for previous observations geometrically. The EWMA statistics are exclusively utilized to analyze shifts in the mean of the process production. Roberts^27^ introduced novel exponentially weighted moving average (EWMA) statistics for the *i*th sample, which has a size of 5, specifically designed for monitoring a single parameter.

1 $$T_{i} = \lambda \overline{X}_{i} + \left( {1 - \lambda } \right)T_{i - 1}$$

Xie^28^ and Chen et al^29^. introduced the concept of integrating the monitoring of both the mean shift and variance shift of parameters into a single control chart, referred to as the Maximum Exponentially Weighted Moving Average (Max-EWMA) control chart. Let X be a normally distributed random variable in process production. It has a mean of $\mu = \mu_{0} + a\sigma_{0}$ and a variance $\sigma^{2} = b^{2} \sigma_{0}^{2}$ here $\mu_{0}$ and $\sigma_{0}^{2}$ are known values for the mean and variance, respectively. The variables b and a represent shifts in variance and mean, respectively. In an in-control method, b has a value of 1 and a has a value of 0. The Max-EWMA statistics have a higher efficiency in detecting minor shifts. The converted statistics for in-control procedures with a zero mean and unit variance for the ith sample follow a normal distribution.

2 $$K_{i} = \frac{{\overline{X}_{i} - \mu_{0} }}{{\sqrt {{\raise0.7ex\hbox{${\sigma_{0}^{2} }$} \!\mathord{\left/ {\vphantom {{\sigma_{0}^{2} } n}}\right.\kern-0pt} \!\lower0.7ex\hbox{$n$}}} }},$$

3 $$L_{i} = \emptyset^{ - 1} \left[ {H\left\{ {\frac{{\left( {n - 1} \right)S_{i}^{2} }}{{\sigma_{0}^{2} }},\left( {n - 1} \right)} \right\}} \right],$$

where i = 1,2,3,…,*n* is the size of *i*th sample, the mean $\overline{X}_{i} = \frac{{\mathop \sum \nolimits_{i = 1}^{n} X_{ij} }}{n}$ of *i*th sample, and variance.

$S_{i}^{2} = \frac{{\mathop \sum \nolimits_{i = 1}^{n} \left( {X_{ij} - \overline{X}_{i} } \right)^{2} }}{n - 1}$ of *i*th sample, $\emptyset^{ - 1}$ is the inverse function of standard normal distribution, while $H\left( {\xi ,v} \right)$ follow $\chi^{2}$ distribution with v degree of freedom.

The two Exponentially Weighted Moving Average (EWMA) statistics can be determined by utilizing Eqs. (2) and (3).

4 $$M_{i} = \lambda K_{i} + \left( {1 - \lambda } \right)K_{i - 1} ,$$

5 $$N_{i} = \lambda L_{i} + \left( {1 - \lambda } \right)L_{i - 1} ,$$

where $M_{0} = N_{0} = 0$ for the first sample and $\lambda$ is a smoothing constant, such that 0 < $\lambda \le 1$.

In the above statistics, the sum of all weights is equal to one. The quantities $M_{i - 1}$ and $N_{i - 1}$ represent the values of the variable in the previous iteration. We will utilize a single maximum absolute statistic to collectively assess both the mean and variance, rather than analyzing each statistic independently.

6 $$K_{i} = {\text{Max}} - {\text{EWMA}} = {\text{ Max }}\left( {\left| {M_{i} } \right|,\left| {N_{i} } \right|} \right)$$

The average and the dispersion of the $K_{i}$ statistic Xie^28^ are provided, correspondingly,

$$E\left( {K_{i} } \right) = \frac{2}{\sqrt \pi } = 1.128379 \;{\text{and}}\;Var\left( {K_{i} } \right) = 1 - \frac{2}{\sqrt \pi } = 0.363380$$

Using Xie^25^ method, it is sufficient to have only one upper control limit (UCL) when plotting Max-EEWMA statistics to control manufacturing methods.

7 $$UCL = \left( {1.128379 + 0.602810 \times L} \right)\sqrt {Var\left( {M_{i} } \right)}$$

The control constant, written as L, is computed to achieve the desired average run length (ARL_0_) for an in-control process and the variance is $Var\left( {M_{i} } \right) = Var\left( {N_{i} } \right) = \sqrt {\frac{\lambda }{{\left( {2 - \lambda } \right)}}}$.

## Proposed MM EWMA control chart for joint monitoring

In this section, the proposed MM EWMA control chart is explained. The quality characteristics ${U}_{i}$ and ${V}_{i}$ are supposed to follow a normal distribution. We suggested the Max Mixed EWMA control chart. Note that a critique paper is presented by Haq and Woodal^30^ on the modified EWMA versions and they specifically discussed the Extended EWMA version of Naveed et al^31^. Aslam et al^32^. presented a rebuttal paper on the use of such modified EWMA statistic. The reader may consult these papers for further clarity. The simple moving averages-based EWMA control chart has an issue in assigning the weights as the statistic assigns more weight to the past observations^33^. This problem occurs as all the observations are assigned equal weights to calculate moving averages. To address this issue, we have implemented a weighted moving averages approach. In this method, 80% of the weight is allocated to the current observation, while the remaining 20% is distributed equally among the previous observations for the EWMA statistic. For a visual representation of the weight distribution to current and past values, reader is referred to Supplementary Figure A and Supplementary Figure B in the Appendix. The design of the proposed control chart is given below:

*Step 1*: Select a sample of *n* size for calculating the weighted moving averages of $U_{i}$ and $V_{i}$ and compute two suggested MM EWMA statistics.

The two suggested MM EWMA statistics for mean, and variance are

8 $$R_{t} = \lambda U_{i} + \left( {1 - \lambda } \right)R_{t - 1} + k\left( {U_{i} - U_{i - 1} } \right),$$

9 $$S_{t} = \lambda V_{i} + \left( {1 - \lambda } \right)S_{t - 1} + k\left( {V_{i} - V_{i - 1} } \right),$$

where $R_{0} = S_{0} = 0$ for the first sample and the smoothing parameter $\lambda$ lies between 0 &$1$ with $k = - \left( {\frac{\lambda }{2}} \right).$

The total weight in the suggested statistics is one. The quantities $U_{i - 1}$ and $V_{i - 1}$ denote the preceding value of the variable, and the $R_{i - 1}$ and $S_{i - 1}$ denote the prior value of the statistic.

The MM EWMA statistics mean and variation for in-control are

10 $$E\left( {R_{t} } \right) = E(S_{t} ) = \mu$$

11 $$Var\left( {R_{t} } \right) = Var\left( {S_{t} } \right) = { }\frac{{\sigma^{2} }}{iw}\left[ {\left( {\frac{{\lambda + 2\lambda k + 2k^{2} }}{2 - \lambda }} \right)} \right]{ }if{ }i < w$$

12 $$Var\left( {R_{t} } \right) = Var\left( {S_{t} } \right) = { }\frac{{\sigma^{2} }}{{w^{2} }}\left[ {\left( {\frac{{\lambda + 2\lambda k + 2k^{2} }}{2 - \lambda }} \right)} \right]{ }if{ }i > w$$

Instead of examining mean and variance individually, we will combine them into a single maximum absolute statistic. The variance proposed chart at the time *t* is calculated from the moving average at each width (*w*).

*Step 2*: Select the Max statistic from the two suggested MMEWMA statistics.

13 $${\text{MM EWMA}} = {\text{ Max}}\;\left( {\left| {R_{t} } \right|,\left| {S_{t} } \right|} \right),$$

where *i* = 1,2, 3, … The maximum statistic available from two absolute statistics for mean and variance is called the MM EWMA statistic.

According to^34^, using absolute quantities only the upper control limit (UCL) is sufficient for plotting MM EWMA statistics for monitoring the manufacturing process.

*Step 3*: The process will be considered in control when the suggested EEWMA statistic exceeds the limit; otherwise, it will be considered out of control. The upper control limit is denoted by “*UCL.*”

14 $$UCL = \left( {1.128379 + 0.602810 \times L} \right)\sqrt {\frac{{\sigma^{2} }}{iw}\left[ {\left( {\frac{{\lambda + 2\lambda k + 2k^{2} }}{2 - \lambda }} \right)} \right]{ }} { }if{ }i < w$$

15 $$UCL = \left( {1.128379 + 0.602810 \times L} \right)\sqrt {\frac{{\sigma^{2} }}{{w^{2} }}\left[ {\left( {\frac{{\lambda + 2\lambda k + 2k^{2} }}{2 - \lambda }} \right)} \right]{ }} { }if{ }i > w$$

The control constant is denoted by *L* and computed to attain *ARL*_*0*_ required average run length for in control process.

## Simulation study

This part evaluates the in-depth analysis of the suggested control chart. The data is generated for this purpose using a normal distribution such that $Y_{i} \sim N\left( {0,1} \right)$. The following steps should be followed to calculate ARL and SDRL for MM EWMA:

*Step 1*: Sample variance and mean control statistics.

i. Compute 30,000 n-size random samples for the in-control process using the normal distribution.
ii. Determine the suggested statistic for each sample.

*Step 2*: Setting up Control Limits.

i. Decide the initial values of two parameters $L$ and $\lambda$.
ii. Compute a statistic that accounts for both variance and mean and is given in (8) and (9).
iii. To obtain MM EWMA statistics, determine the statistic for both mean and variance given in (13).
iv. Following the design of the control chart, examine the proposed statistic in the presence of an out-of-control signal. When the process is considered in-control, go on to step v. If not, note the number of samples as the run length for in-control.
v. To evaluate *ARL*_*0*_, repeat steps ii through iv 30,000 times. If we reach the required *ARL*_*0*_, go to Step 3 with the current value of $L$. If not, modify the value of $L$ and repeat steps ii through v in Step 2.

*Step 3*: Calculation of out-of-control* ARL.*

i. Produce a random variable *Y* from a normal distribution with a mean shift for each sample, denoted as $Y\sim N\left(a, 1*b\right)$, where in control mean and variance are considered as zero and one respectively. Here, *a* is the shift in mean and *b* is the shift in variance.
ii. Analyze the sample with the proposed statistic.
iii. The process will continue to cycle through steps i and ii from step 3 until the process is no longer in control. A run length will be recorded based on the total number of samples taken.
iv. To determine an accurate number for ARL and SDRL, we shall repeat this process 30,000 times. We followed the design of the control chart described in section "Proposed MM EWMA Control Chart for Joint Monitoring" to obtain the out-of-control run lengths.
v. If MM EWMA stays within the UCL's region, the process will be in-control. After that, make another sample and go back through the first four steps again.
vi. If the MM EWMA statistic falls outside the UCL limit, the procedure is now considered to be out-of-control, production ends, and the target run length is reached. But in the case of steady state ARL cut point is also considered.

The 30,000 replicates were used to compute each ARL, SDRL, and MRL (median run length). In this study, *a*=0.0,0.05, 0.1, 0.25, 0.50, 0.75, 1, 1.5, 2.0, and *b*=0.25, 0.50, 0.75, 0.90, 1.00, 1.10, 1.25, 1.50, 2.00, 2.50, 3.00 were utilized in various combinations for mean shift and variance shift, respectively. Tables 1, 2 show that the MM EWMA chart is effective at simultaneously detecting the shifts in mean and variance. Supplementary Table 2 shows the steady-state ARLs of the proposed control chart. The ability to identify both changes at an early stage is demonstrated by the ease with which ARLs degrade as mean shift increases and, similarly, with increases in variance shift.

**Table 1:** *ARL*, *SDRL* & *MRL* of MM EWMA chart and Max-EWMA chart for $\lambda$ =0.20 & ARL_o_ = 370.

		MM EWMA				MM EWMA			Max-EWMA		
Shift		$\lambda$= 0.20, w = 2				$\lambda$= 0.20, w = 3			$\lambda$=0.20		
		CL = 0.9301				CL = 0.9112			L = 3.254		
*b*	*a*	ARL	SDRL	MRL		ARL	SDRL	MRL	ARL	SDRL	MRL
0.25	0	2.81	0.37	3		2.81	0.47	3	2.82	0.5	3
	0.05	2.81	0.36	3		2.81	0.47	3	2.82	0.5	3
	0.1	2.8	0.37	3		2.8	0.48	3	2.82	0.49	3
	0.25	2.8	0.37	3		2.8	0.47	3	2.82	0.5	3
	0.5	2.8	0.36	3		2.79	0.47	3	2.82	0.49	3
	0.75	2.79	0.36	3		2.79	0.47	3	2.82	0.5	3
	1	2.77	0.28	3		2.77	0.36	3	2.78	0.44	3
	1.5	2	0.36	2		2	0.26	2	2	0.05	2
	2	2	0	2		2	0	2	2	0.06	2
0.5	0	5.57	1.67	5		5.38	1.73	5	5.79	1.93	5
	0.05	5.56	1.67	5		5.39	1.73	5	5.78	1.93	5
	0.1	5.57	1.67	5		5.38	1.72	5	5.79	1.94	5
	0.25	5.56	1.66	5		5.39	1.71	5	5.78	1.92	5
	0.5	5.4	1.44	5		5.15	1.48	5	5.62	1.71	5
	0.75	4.41	0.81	4		4.22	0.9	4	4.46	0.96	4
	1	3.24	0.54	3		3.27	0.56	3	3.29	0.58	3
	1.5	2.07	0.25	2		2.06	0.29	2	2.08	0.27	2
	2	1.9	0.04	2		1.91	0.08	2	1.91	0.28	2
0.75	0	26.49	20.1	21		25.61	19.8	20	29.52	22.94	23
	0.05	26.33	19.89	20		25.45	19.62	20	29.55	23.07	23
	0.1	26.11	19.75	20		25.2	19.11	20	29.19	22.73	22
	0.25	21.74	15.22	17		20.97	14.98	17	24.47	17.84	19
	0.5	8.73	3.8	8		8.32	3.86	8	9.41	4.38	8
	0.75	4.86	1.39	5		4.68	1.46	4	4.92	1.57	5
	1	3.24	0.55	3		3.37	0.82	3	3.37	0.86	3
	1.5	2.15	0.39	2		2.15	0.43	2	2.17	0.4	2
	2	1.81	0.15	2		1.81	0.21	2	1.82	0.39	2
0.85	0	93.92	87.1	67		92.01	86.69	65	105.53	98.27	75
	0.05	91.96	85.08	66.5		90.01	84.3	64	103.7	96.54	74
	0.1	84.13	76.78	61		81.02	75.57	58	94.29	87.5	67
	0.25	35.9	29.01	27		34.54	28.61	26	40.34	33.37	30
	0.5	9.15	4.52	8		8.76	4.53	8	9.65	5.01	8
	0.75	4.95	1.62	5		4.78	1.69	5	4.97	1.79	5
	1	3.37	0.84	3		3.4	0.92	3	3.4	0.97	3
	1.5	2.19	0.45	2		2.2	0.46	2	2.2	0.46	2
	2	1.74	0.19	2		1.78	0.25	2	1.79	0.42	2
0.9	0	198.96	191.66	141		194.33	191.5	136	216.49	210.17	152
	0.05	184.12	176.19	130		179.49	176.86	125	201.55	194.98	142
	0.1	145.57	138.91	103		142.6	136.87	101	160.53	154.6	113
	0.25	39.04	32.68	29		37.44	31.82	28	42.76	36.19	32
	0.5	9.15	4.73	8		8.87	4.85	8	9.62	5.18	8
	0.75	4.97	1.69	5		4.82	1.76	5	4.97	1.86	5
	1	3.29	0.9	3		3.4	1	3	3.41	1.02	3
	1.5	2.2	0.44	2		2.2	0.48	2	2.21	0.48	2
	2	1.75	0.21	2		1.77	0.27	2	1.78	0.43	2
1	0	370.94	365.68	256		370.24	366.98	257	370.32	366.98	257
	0.05	294.94	288.81	207		291.06	291.41	201	300.73	295.87	210
	0.1	174.45	169.81	123		173.05	169.64	122	182.23	177.51	128
	0.25	36.08	30.35	27		34.39	29.82	25	37.67	31.93	28
	0.5	9.16	4.98	8		8.81	5.08	8	9.41	5.32	8
	0.75	4.95	1.86	5		4.87	1.98	5	4.98	2.01	5
	1	3.41	1	3		3.4	1.1	3	3.43	1.13	3
	1.5	2.2	0.52	2		2.21	0.51	2	2.22	0.53	2
	2	1.67	0.25	2		1.76	0.31	2	1.76	0.45	2
1.05	0	200.42	196.01	142		199.9	197.62	139	200.45	196.06	140
	0.05	173.32	167.9	121		171.68	170.23	120	174.27	170.15	122
	0.1	119.8	114.42	85		117.15	114.93	82	121.82	116.72	86
	0.25	32.24	26.68	24		30.81	26.22	23	33.31	27.9	25
	0.5	9.09	5.08	8		8.68	5.13	7	9.27	5.34	8
	0.75	4.94	1.95	5		4.85	2.02	4	4.98	2.11	5
	1	3.43	1.06	3		3.44	1.16	3	3.44	1.17	3
	1.5	2.22	0.52	2		2.22	0.52	2	2.23	0.55	2
	2	1.72	0.27	2		1.75	0.32	2	1.75	0.46	2
1.1	0	95.74	90.69	69		95.13	90.68	67	95.85	90.92	68
	0.05	88.75	83.01	64		86.69	83.66	61	89.28	84.02	64
	0.1	70.98	65.52	51		69.79	66.08	49	71.25	66.17	51
	0.25	27.57	22.14	21		26.29	22.18	20	27.94	22.96	21
	0.5	8.93	5.06	8		8.52	5.09	7	8.98	5.19	8
	0.75	4.96	2.03	5		4.87	2.12	4	4.97	2.16	5
	1	3.42	1.11	3		3.43	1.21	3	3.44	1.21	3
	1.5	2.23	0.58	2		2.23	0.55	2	2.24	0.59	2
	2	1.69	0.28	2		1.74	0.34	2	1.75	0.48	2
1.15	0	50.19	45.29	37		49.64	45.91	36	50.66	45.93	37
	0.05	47.95	42.94	35		46.4	42.97	34	48.39	43.47	35
	0.1	42.91	37.89	32		41.37	37.9	30	43.2	38.35	32
	0.25	22.25	17.65	17		21.48	17.66	16	22.52	17.75	17
	0.5	8.66	4.91	7		8.32	5.05	7	8.67	5.06	7
	0.75	4.94	2.09	5		4.85	2.17	4	4.95	2.19	5
	1	3.45	1.15	3		3.43	1.26	3	3.46	1.26	3
	1.5	2.24	0.6	2		2.23	0.61	2	2.25	0.61	2
	2	1.7	0.3	2		1.74	0.36	2	1.75	0.49	2
1.25	0	20.72	16.18	16		19.99	16.33	15	20.8	16.43	16
	0.05	20.37	16.02	16		19.55	16.04	15	20.38	16.06	16
	0.1	19.42	14.95	15		18.63	14.89	14	19.43	14.99	15
	0.25	14.42	10.34	12		13.88	10.33	11	14.56	10.4	12
	0.5	7.72	4.41	7		7.48	4.42	6	7.78	4.42	7
	0.75	4.27	2.13	4		4.77	2.14	4	4.82	2.22	4
	1	3.37	1.23	3		3.43	1.24	3	3.44	1.32	3
	1.5	2.25	0.63	2		2.24	0.61	2	2.26	0.66	2
	2	1.72	0.32	2		1.74	0.39	2	1.74	0.52	2
1.5	0	6.95	4.1	6		6.82	4.1	6	7.05	4.14	6
	0.05	6.99	4.03	6		6.78	4.01	6	7.02	4.08	6
	0.1	6.86	4.01	6		6.72	4.03	6	6.94	4.05	6
	0.25	6.41	3.63	6		6.28	3.59	5	6.52	3.66	6
	0.5	5.23	2.67	5		5.21	2.69	5	5.31	2.7	5
	0.75	4.08	1.82	4		4.12	1.84	4	4.14	1.89	4
	1	3.13	1.27	3		3.21	1.31	3	3.26	1.33	3
	1.5	2.17	0.69	2		2.23	0.76	2	2.26	0.77	2
	2	1.66	0.38	2		1.71	0.45	2	1.73	0.57	2
2	0	3.05	1.22	3		3.12	1.26	3	3.13	1.42	3
	0.05	3.1	1.31	3		3.14	1.42	3	3.15	1.43	3
	0.1	3.09	1.37	3		3.1	1.4	3	3.13	1.42	3
	0.25	3.03	1.33	3		3.07	1.35	3	3.1	1.4	3
	0.5	2.88	0.98	3		2.94	1.1	3	2.96	1.3	3
	0.75	2.67	1	3		2.74	1.15	2	2.75	1.17	3
	1	2.49	0.86	3		2.51	1.02	2	2.51	1.03	2
	1.5	2.02	0.61	2		2.02	0.77	2	2.06	0.79	2
	2	1.68	0.39	2		1.69	0.48	2	1.69	0.64	2
2.5	0	2.09	0.75	2		1.75	0.77	2	2.16	0.92	2
	0.05	2.13	0.77	2		1.33	0.78	2	2.16	0.92	2
	0.1	2.07	0.78	2		2.14	0.81	2	2.15	0.92	2
	0.25	2.1	0.8	2		1.9	0.7	2	2.14	0.91	2
	0.5	2.03	0.73	2		2.1	0.68	2	2.1	0.89	2
	0.75	2.02	0.83	2		1.33	0.82	2	2.04	0.85	2
	1	1.93	0.43	2		1.93	0.57	2	1.95	0.8	2
	1.5	1.72	0.61	2		1.73	0.69	2	1.75	0.71	2
	2	1.47	0.34	2		1.52	0.49	2	1.54	0.61	1
3	0	1.6	0.59	2		1.5	0.65	2	1.69	0.72	2
	0.05	1.69	0.63	2		1.5	0.67	2	1.7	0.72	2
	0.1	1.65	0.42	2		1.5	0.68	2	1.69	0.71	2
	0.25	1.62	0.52	2		1.5	0.68	2	1.69	0.71	2
	0.5	1.6	0.69	2		1.4	0.68	2	1.67	0.7	2
	0.75	1.61	0.66	2		1.63	0.59	2	1.64	0.69	2
	1	1.54	0.54	2		1.6	0.6	2	1.6	0.66	2
	1.5	1.47	0.57	2		1.5	0.59	2	1.51	0.61	1
	2	1.34	0.53	2		1.38	0.52	2	1.39	0.55	1

**Table 2:** *ARL*, *SDRL* & *MRL* of MM EWMA chart and Max-EWMA chart for $\lambda$ =0.30 & ARL_o_ = 370.

		MM EWMA				MM EWMA			Max-EWMA		
Shift		$\lambda$= 0.30, w = 2				$\lambda$= 0.30, w = 3			$\lambda$=0.30		
		CL = 1.1543				CL = 1.1247			L = 3.346		
*b*	*a*	ARL	SDRL	MRL		ARL	SDRL	MRL	ARL	SDRL	MRL
0.25	0	2.53	0.46	3		2.52	0.53	3	2.53	0.55	3
	0.05	2.53	0.46	3		2.51	0.53	3	2.53	0.55	3
	0.1	2.52	0.46	3		2.5	0.53	3	2.54	0.55	3
	0.25	2.52	0.46	3		2.5	0.53	3	2.53	0.55	3
	0.5	2.51	0.46	3		2.49	0.53	3	2.53	0.55	3
	0.75	2.51	0.46	3		2.49	0.52	3	2.54	0.55	3
	1	2.48	0.43	3		2.47	0.48	3	2.49	0.51	2
	1.5	2	0.05	2		1.99	0.07	2	2	0.04	2
	2	1.35	0	2		1.38	0.01	2	1.39	0.49	1
0.5	0	5.5	2.14	5		5.21	2.14	5	6.16	2.78	6
	0.05	5.49	2.15	5		5.23	2.15	5	6.19	2.74	6
	0.1	5.5	2.15	5		5.22	2.15	5	6.13	2.75	6
	0.25	5.48	2.14	5		5.2	2.11	5	6.19	2.81	6
	0.5	5.28	1.83	5		4.94	1.82	5	5.94	2.47	5
	0.75	4.08	0.92	4		3.82	0.96	4	4.37	1.23	4
	1	3.04	0.48	3		3.02	0.59	3	3.05	0.66	3
	1.5	2	0.29	2		1.98	0.21	2	2	0.19	2
	2	1.44	0	2		1.43	0.02	2	1.45	0.5	1
0.75	0	37.68	32.77	28		35.29	31.35	26	47.11	42.2	34
	0.05	37.59	32.7	28		35.22	31.29	26	47.19	42.52	34
	0.1	37.32	32.49	27		35.09	31.49	26	46.43	41.3	34
	0.25	30.91	25.9	23		28.79	25.05	21	39.39	34.51	29
	0.5	9.7	5.58	8		9.01	5.54	8	11.55	7.34	10
	0.75	4.63	1.64	4		4.37	1.66	4	4.93	2.01	5
	1	3.14	0.79	3		3.11	0.86	3	3.15	0.96	3
	1.5	2	0.4	2		1.99	0.43	2	2	0.41	2
	2	1.44	0.05	2		1.45	0.1	2	1.46	0.5	1
0.85	0	136.59	130.92	96		130.65	127.69	91	163.9	159.56	115
	0.05	134.09	129.39	95		128.87	126.75	90	158.98	154.48	112
	0.1	123.13	119.47	86		117.84	115.16	82	148.42	145.15	104
	0.25	52.11	46.95	38		48.98	45.19	35	65.46	60.52	47
	0.5	10.11	6.31	8		9.37	6.08	8	11.5	7.59	9
	0.75	4.71	1.87	4		4.48	1.9	4	4.94	2.22	4
	1	3.16	0.9	3		3.16	0.97	3	3.17	1.07	3
	1.5	2	0.42	2		2	0.46	2	2.01	0.47	2
	2	1.44	0.08	2		1.46	0.14	2	1.47	0.5	1
0.9	0	262.68	258.09	183		254.23	253.51	175	296.6	293.5	208
	0.05	244.14	239.78	170		237.7	235.81	165	281.4	277.04	197
	0.1	197.63	191.36	140		190.84	189.1	133	228.76	223.56	160
	0.25	54.68	50.02	39		51.14	48.22	36	65.46	61.16	47
	0.5	9.93	6.36	8		9.31	6.23	8	11.25	7.65	9
	0.75	4.74	1.96	4		4.52	1.99	4	4.94	2.28	4
	1	3.16	0.95	3		3.19	1.02	3	3.19	1.13	3
	1.5	2	0.44	2		1.99	0.48	2	2	0.51	2
	2	1.44	0.1	2		1.47	0.16	2	1.48	0.5	1
1	0	370.6	369.8	257		370.79	369.87	253	370.36	367.18	259
	0.05	312.2	309.96	217		310.69	309.97	215	320.31	318.54	222
	0.1	205.93	202.52	144		200.69	199.42	139	217.98	213.97	153
	0.25	45.5	41.3	33		42.7	40.26	31	51.27	47.28	37
	0.5	9.66	6.3	8		9.08	6.27	7	10.53	7.15	9
	0.75	4.78	2.15	4		4.55	2.16	4	4.92	2.4	4
	1	3.17	1.06	3		3.21	1.12	3	3.21	1.22	3
	1.5	2	0.47	2		2	0.52	2	2.01	0.57	2
	2	1.45	0.13	2		1.46	0.21	2	1.47	0.51	1
1.05	0	208.9	204.18	147		208.59	207.19	144	209	204.08	147
	0.05	186.47	182.41	131		182.78	181.23	126	188.13	185.15	130
	0.1	135.68	132.72	95		130.83	129.32	90	138.97	135.31	97
	0.25	38.41	34.24	28		36.77	34.42	26	41.81	38.2	30
	0.5	9.5	6.3	8		8.95	6.26	7	10.07	6.89	8
	0.75	4.81	2.23	4		4.56	2.21	4	4.89	2.48	4
	1	3.18	1.12	3		3.2	1.17	3	3.21	1.27	3
	1.5	2	0.48	2		2	0.55	2	2.01	0.59	2
	2	1.44	0.15	2		1.47	0.22	2	1.48	0.51	1
1.1	0	105.42	101.7	75		102.65	101.21	71	105.44	101.86	74
	0.05	97.89	93.88	69		95.89	94.9	66	99.66	96.29	70
	0.1	80.99	77.06	58		78.3	77.26	55	82.08	78.9	58
	0.25	31.93	28.2	23		30.11	27.83	22	33.12	29.67	24
	0.5	9.25	6.14	8		8.58	6.05	7	9.67	6.64	8
	0.75	4.8	2.27	4		4.54	2.28	4	4.86	2.49	4
	1	3.18	1.16	3		3.2	1.21	3	3.21	1.32	3
	1.5	2	0.5	2		2	0.56	2	2.01	0.62	2
	2	1.45	0.17	2		1.47	0.25	2	1.49	0.52	1
1.15	0	57.24	54.1	41		54.58	54.11	38	57.64	54.69	41
	0.05	54.8	50.98	39		52.22	50.7	37	55.13	52.06	39
	0.1	48.08	44.51	35		45.45	44.15	32	48.87	45.44	35
	0.25	24.99	21.44	19		23.58	21.43	17	25.65	22.18	19
	0.5	8.87	5.89	7		8.29	5.91	7	9.08	6.13	7
	0.75	4.79	2.31	4		4.54	2.36	4	4.79	2.5	4
	1	3.19	1.21	3		3.21	1.27	3	3.22	1.35	3
	1.5	2.01	0.52	2		2.01	0.58	2	2.02	0.65	2
	2	1.44	0.19	2		1.47	0.26	2	1.49	0.52	1
1.25	0	22.61	19.44	17		20.9	19.02	15	22.64	19.25	17
	0.05	22	18.74	16		20.63	19.03	15	22.02	18.81	16
	0.1	21.05	18.01	16		19.59	17.66	14	21.1	18.06	16
	0.25	15.43	12.28	12		14.27	12.1	11	15.44	12.39	12
	0.5	7.8	5.01	6		7.24	5.05	6	7.89	5.25	6
	0.75	4.58	2.32	4		4.41	2.34	4	4.63	2.49	4
	1	3.19	1.26	3		3.19	1.32	3	3.19	1.4	3
	1.5	2.01	0.55	2		2.01	0.61	2	2.02	0.7	2
	2	1.44	0.22	2		1.48	0.29	2	1.49	0.53	1
1.5	0	6.78	4.52	6		6.4	4.51	5	6.83	4.54	6
	0.05	6.77	4.52	6		6.42	4.56	5	6.81	4.55	6
	0.1	6.71	4.46	6		6.27	4.43	5	6.74	4.5	6
	0.25	6.23	3.99	5		5.89	4.05	5	6.25	4	5
	0.5	4.96	2.84	4		4.8	2.94	4	4.98	2.92	4
	0.75	3.8	1.91	4		3.78	1.93	3	3.81	1.99	3
	1	2.91	1.27	3		2.97	1.31	3	2.97	1.37	3
	1.5	1.97	0.62	2		1.99	0.68	2	2	0.78	2
	2	1.5	0.31	2		1.5	0.38	2	1.51	0.57	1
2	0	2.82	1.24	3		2.8	1.43	3	2.83	1.45	3
	0.05	2.79	1.08	3		2.79	1.4	3	2.81	1.45	2
	0.1	2.76	1.19	3		2.79	1.37	3	2.83	1.44	3
	0.25	2.64	0.72	3		2.75	1.33	2	2.77	1.41	2
	0.5	2.54	1.09	2		2.6	1.26	2	2.63	1.31	2
	0.75	2.36	0.79	2		2.39	1.04	2	2.42	1.15	2
	1	2.15	0.9	2		2.16	0.84	2	2.22	1.02	2
	1.5	1.76	0.58	2		1.78	0.61	2	1.81	0.78	2
	2	1.44	0.41	2		1.45	0.48	2	1.47	0.6	1
2.5	0	1.67	0.45	2		1.89	0.53	2	1.91	0.89	2
	0.05	1.88	0.48	2		1.88	0.74	2	1.9	0.89	2
	0.1	1.67	0.81	2		1.88	0.86	2	1.9	0.89	2
	0.25	1.74	0.87	2		1.8	0.74	2	1.88	0.88	2
	0.5	1.75	0.85	2		1.8	0.83	2	1.85	0.86	2
	0.75	1.72	0.73	2		1.5	0.7	2	1.78	0.82	2
	1	1.68	0.75	2		1.5	0.7	2	1.71	0.77	2
	1.5	1.5	0.61	2		1.25	0.65	2	1.54	0.66	1
	2	1.35	0.35	2		1.33	0.63	2	1.37	0.55	1
3	0	1.5	0.45	2		1.5	0.64	2	1.51	0.67	1
	0.05	1.5	0.61	2		1.49	0.62	2	1.5	0.66	1
	0.1	1.48	0.5	2		1.33	0.63	2	1.5	0.66	1
	0.25	1.47	0.54	2		1.33	0.61	2	1.5	0.66	1
	0.5	1.46	0.58	2		1.4	0.6	1	1.49	0.65	1
	0.75	1.41	0.57	2		1.42	0.61	2	1.46	0.63	1
	1	1.4	0.54	2		1.33	0.58	2	1.42	0.6	1
	1.5	1.31	0.52	2		1.25	0.53	2	1.34	0.55	1
	2	1.21	0.46	2		1	0.69	1	1.26	0.48	1

## Illustration example

The proposed chart’s implementation is evaluated using the data set given by^35^ and^36^. The 200 values from the yogurt packing process in a cup with 125 g of the quality characteristic "X" are included in the data. Following the long-term phase-I research, the in-control parameters' mean, and standard deviation were calculated to be ${\mu }_{o}$ = 124.9 and ${\sigma }_{o}=$ 0.76, respectively. Every hour, the quality control experts select a sample and record its weight twice to maintain the consistency of the cup filling. The 20 samples of size 5 that were generated in the aforementioned papers are still used in this article, but we consider the first 100 data values to be in control and the remaining 100 values to be shifted. We specify the design parameters employed in constructing the control chart when the ARL_0_ = 370. We consider the selection of the smoothing constant as λ = 0.3, which determines the degree of smoothing applied to the data. Additionally, the parameter *w* = 3 is taken that signifies the width of the moving average window used for smoothing. Note that the value of smoothing constant is 0.3 for the Max-EWMA control chart taking ARL_0_ = 370. Then, we randomly select 20 samples, 10 from within the control range and the remaining 10 from the shifted range, for each of the five sizes of yogurt cups. We then used these 20 values to derive the proposed MM EWMA statistic and UCL. Table 3 provides sample statistics and upper control limits.

**Table 3:** MM EWMA statistics and Max-EWMA statistics for the yogurt filling data set.

Sample	MM EWMA	*UCL*	MAX-EWMA	*UCL*_*1*_
1	0.1134357	1.6203	0.2268715	1.35627
2	0.2905495	1.6203	0.1092387	1.35627
3	0.2640641	1.6203	0.1407251	1.35627
4	0.2042986	1.6203	0.1627656	1.35627
5	0.2164639	1.6203	0.178194	1.35627
6	0.1835939	1.6203	0.1889938	1.35627
7	0.1927738	1.6203	0.1965537	1.35627
8	0.1991997	1.6203	0.2018456	1.35627
9	0.3237746	1.6203	0.567394	1.35627
10	0.5222057	1.6203	0.5864813	1.35627
11	0.6076328	1.6203	0.623734	1.35627
12	0.9639446	1.6203	1.0563177	1.35627
13	1.4433872	1.6203	1.1538552	1.35627
14	1.992385	1.6203	1.9788169	1.35627
15	2.4533583	1.6203	2.3416275	1.35627
16	2.7935479	1.6203	2.8652648	1.35627
17	2.9969322	1.6203	2.9312833	1.35627
18	3.0935513	1.6203	3.1881339	1.35627
19	3.2488608	1.6203	3.0580103	1.35627
20	3.3890506	1.6203	3.5196798	1.35627

**Figure 1:**
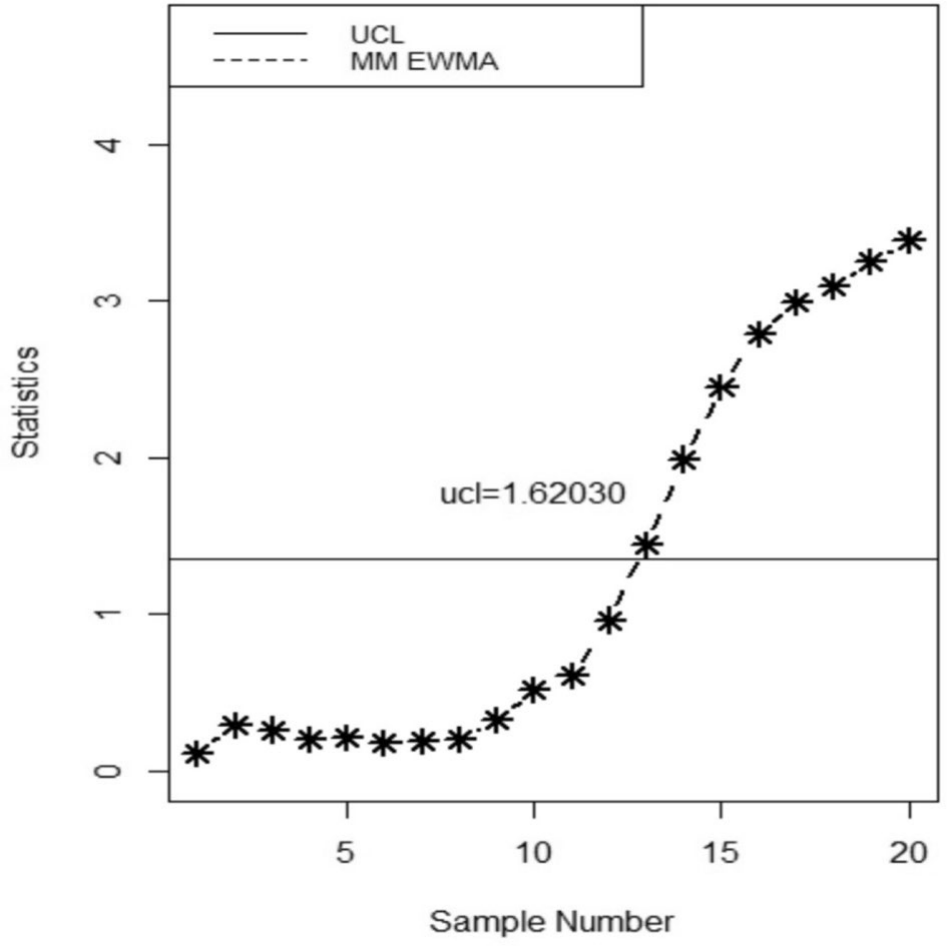
MM EWMA control chart.

**Figure 2:**
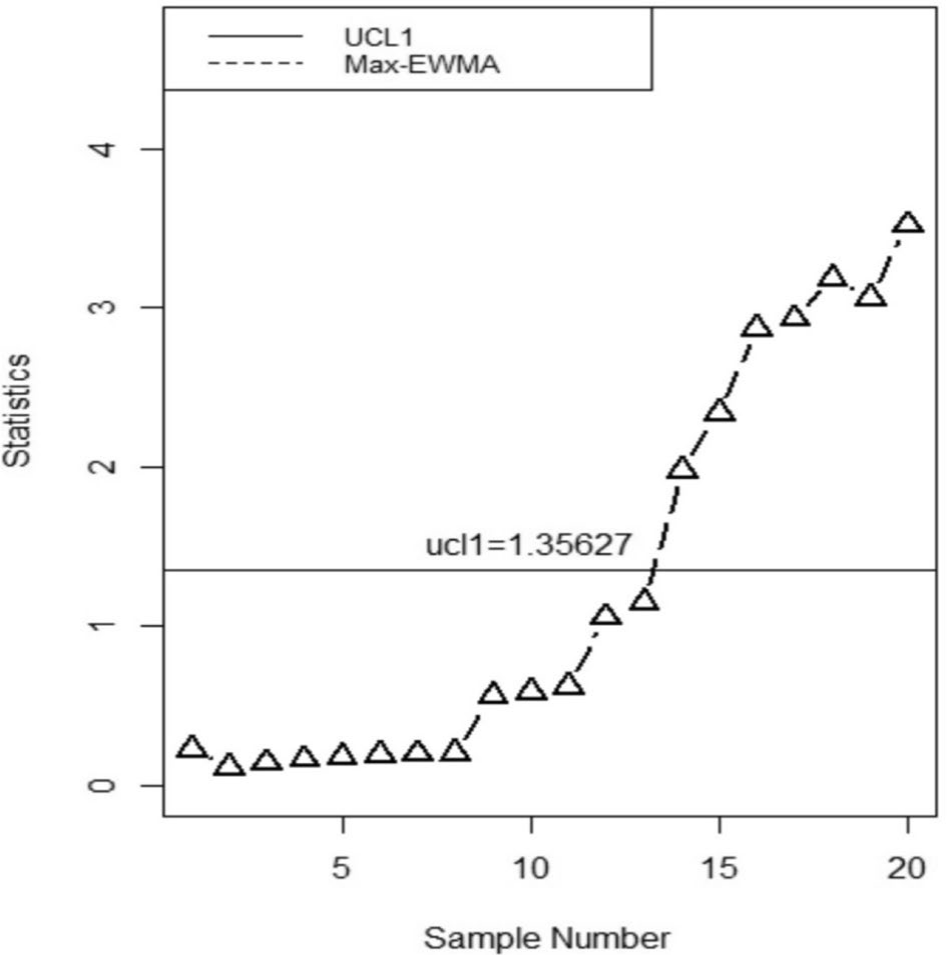
Max-EWMA control chart.

Figure 1 presents a visually consistent representation of the data from Table 3, which illustrates the MM EWMA control chart suggested in this study. The suggested chart is denoted as UCL in the provided chart, whereas the existing chart is represented as UCL1 in Fig. 2, depicted as a horizontal line. The proposed chart clearly demonstrates an out-of-control signal at the 13th value, whereas the existing chart reveals this signal at the 14th sample. Hence, it is evident from the analysis of Figs. 1, 2 that the chart provided in this study exhibited superior performance compared to the previous chart.

## Main findings

MM EWMA control chart results are depicted in Tables 1, 2, The *ARLs* and *SDRLs* for $\lambda$ **=** 0.2 are presented in Table 1 for different values of *w* and Table 2 for $\lambda$= 0.3. The results of MM EWMA statistics are presented in Table 3 with Max-EWMA statistics. The main results of the proposed chart are mentioned below.

The process is without shift when *a* = 0 and *b* = 1, and it signifies that the procedure is in control with 370 *ARL*_*0*_. It can be observed from all three tables that when mean shifts "*a*" increases from 0.00 to 0.10, 0.25, 0.50, 0.75, 1.00, 1.50, and 2.00, the respective values of *ARLs* and *SDRLs* decrease. It demonstrates that the suggested chart is more effective in detecting mean shifts early. The variance shift "*b*" exhibits the same pattern as "*a*" The values of ARLs & SDRLs are decreased in accordance with variations in mean shift "*a*" as the variance shift changes from 1 to 0.25, 0.50, 0.75, 0.90, and from 1 to 1.10, 1.25, 1.50, 2.00, 2.50, and 3.00. Additionally, variance shift is also quickly detected, which further illustrates the effectiveness of the proposed chart. The efficiency of our proposed Max- Mixed EWMA control chart for the combined detection of mean and variance shifts is revealed by the last column in all Tables 1, 2, which compares it to the present Max-EWMA control chart in terms of ARLs. As values of w increase from 2 to 3 for different values of $\lambda$ **=** 0.2, and 0.3, the *ARLs* and *SDRLs* decrease. In Table 2, when *w* = 2, the value of *ARL* is 197.63 for *b* = 0.90 and *a* = 0.1, for *w* = 3, *ARL* is 190.84 on same values of *a* and *b*. The expected values of the *ARLs* are presented in supplementary Table 1 which shows the efficiency of the proposed control chart. Supplementary Table 2 shows the steady-state ARLs. The pattern of steady-state ARLs is the same as expected. The real life application is capable of detecting out-of-control single earlier than the existing chart. This finding is also supported by the results of simulations, as evident from the observations made in Figs. 1 and 2. Specifically, the proposed chart identify out of control at the 13th value, whereas the existing chart detects them at the 14th value.

From the above results and data from calculations in the *ARL* values tables, we can infer that our developed MM EWMA control chart works better than existing control charts. Even smaller mean and variance shifts are effectively detected by the suggested chart simultaneously. Instead of using two separate statistics for mean and variance, the proposed control chart allows for simultaneously investigating both mean and variance process shifts with one statistic.

## Conclusion

Several types of control charts have been developed by different researchers in the quality control field, but many researchers ignored the joint monitoring of mean and variance simultaneously. In this article, we addressed the issue of joint monitoring and developed a new control chart named the MM EWMA control chart. The values of ARLs and SDRLs have been calculated, and tables were created for multiple smoothing constant values with different mean and variance shifts. The efficacy of our proposed MM EWMA control chart for simultaneously detecting shifts in both mean and variance is demonstrated by the final column in all tables. It compares the performance of the proposed chart to that of the current Max-EWMA control chart. Comparisons to the existing Max-EWMA control chart were made regarding ARLs and SDRLs, revealing that the proposed chart identifies mean and variance shifts quicker than the Max-EWMA chart. The real-life application of the MM EWMA control chart was also illustrated, and it also shows the efficiency of the developed chart. The real-life application shows the individual instances that are out-of-control earlier than the current application. The developed control chart can be used in manufacturing for joint monitoring of shifts in the mean and variance during production.

### Supplementary Information

Supplementary Information.

## Data Availability

The datasets used and/or analyzed during the current study are available from the corresponding author upon reasonable request.
