# Supplementary material for: Max-mixed EWMA control chart for joint monitoring of mean and variance: an application to yogurt packing process
Source: Sci Rep. 2024 May 6;14:10372. doi: 10.1038/s41598-024-61132-0 (PMC11074330; doi:10.1038/s41598-024-61132-0)
Supplement: Supplementary file 1 — Supplementary Information. [file 41598_2024_61132_MOESM1_ESM.docx]

**Appendix:**

Supplementary Table 1**:** Expected values of *ARL*, *SDRL* & *MRL* of MM EWMA and Max-EWMA charts for $\lambda$ =0.30 & ARL_o_ = 370.

|  | | MM EWMA | | | MM EWMA | | | | Max-EWMA | | |
| --- | --- | --- | --- | --- | --- | --- | --- | --- | --- | --- | --- |
| **Shift** | | $\lambda$ **= 0.30, w=2** | | | $\lambda$ **= 0.30, w=3** | | | | $\lambda$ **=0.30** | | |
|  |  | **CL = 1.1543** | | | **CL = 1.1247** | | | | **L=3.346** | | |
| ***b*** | ***a*** | **ARL** | **SDRL** | **MRL** | | **ARL** | **SDRL** | **MRL** | **ARL** | **SDRL** | **MRL** |
| 0.75 | 0 | 37.65 | 32.75 | 28 | | 35.21 | 31.33 | 26 | 47.09 | 42.19 | 34 |
|  | 0.1 | 37.29 | 32.43 | 27 | | 35.04 | 31.43 | 26 | 46.33 | 41.18 | 34 |
|  | 1 | 3.08 | 0.73 | 3 | | 3.08 | 0.84 | 3 | 3.12 | 0.94 | 3 |
| 0.85 | 0 | 136.55 | 130.89 | 96 | | 130.61 | 127.65 | 91 | 163.88 | 159.54 | 115 |
|  | 0.1 | 123.11 | 119.44 | 86 | | 117.79 | 115.11 | 82 | 148.39 | 145.13 | 104 |
|  | 1 | 3.15 | 0.88 | 3 | | 3.11 | 0.94 | 3 | 3.13 | 1.03 | 3 |
| 0.9 | 0 | 262.61 | 258.02 | 183 | | 254.19 | 253.48 | 175 | 296.57 | 293.48 | 208 |
|  | 0.1 | 197.59 | 191.31 | 140 | | 190.73 | 189.07 | 133 | 228.69 | 223.51 | 160 |
|  | 1 | 3.13 | 0.92 | 3 | | 3.15 | 1.01 | 3 | 3.17 | 1.06 | 3 |
| 1 | 0 | 370.49 | 369.75 | 257 | | 370.61 | 369.71 | 253 | 370.26 | 367.08 | 259 |
|  | 0.1 | 205.89 | 202.48 | 144 | | 200.55 | 199.31 | 139 | 217.89 | 213.87 | 153 |
|  | 1 | 3.15 | 1.04 | 3 | | 3.11 | 1.03 | 3 | 3.16 | 1.17 | 3 |
| 1.05 | 0 | 208.82 | 204.17 | 147 | | 208.48 | 207.09 | 144 | 208.95 | 204.04 | 147 |
|  | 0.1 | 135.64 | 132.71 | 95 | | 130.71 | 129.23 | 90 | 138.91 | 135.29 | 97 |
|  | 1 | 3.16 | 1.11 | 3 | | 3.11 | 1.12 | 3 | 3.16 | 1.19 | 3 |
| 1.15 | 0 | 57.22 | 54.08 | 41 | | 54.47 | 54.01 | 38 | 57.59 | 54.63 | 41 |
|  | 0.1 | 48.05 | 44.48 | 35 | | 45.36 | 44.04 | 32 | 48.82 | 45.41 | 35 |
|  | 1 | 3.17 | 1.19 | 3 | | 3.14 | 1.25 | 3 | 3.16 | 1.32 | 3 |
| 1.5 | 0 | 6.76 | 4.51 | 6 | | 6.36 | 4.43 | 5 | 6.79 | 4.49 | 6 |
|  | 0.1 | 6.69 | 4.44 | 6 | | 6.21 | 4.36 | 5 | 6.70 | 4.43 | 6 |
|  | 1 | 2.90 | 1.26 | 3 | | 2.91 | 1.28 | 3 | 2.96 | 1.36 | 3 |

Supplementary Table 2**: Steady-State** *ARL*, *SDRL* & *MRL* of MM EWMA chart for $\lambda$ = 0.20, *w* = 3 & ARL_o_ = 370.

| **b** | **a** | **Steady State ARL: 100** | | | **Steady State ARL: 50** | | | **Steady State ARL: 20** | | | **Steady State ARL: 10** | | |
| --- | --- | --- | --- | --- | --- | --- | --- | --- | --- | --- | --- | --- | --- |
|  |  | **ARL** | **SDRL** | **MRL** | **ARL** | **SDRL** | **MRL** | **ARL** | **SDRL** | **MRL** | **ARL** | **SDRL** | **MRL** |
| 0.25 | 0 | 3.018 | 0.497 | 3 | 3.022 | 0.487 | 3 | 3.024 | 0.472 | 3 | 3.029 | 0.468 | 3 |
|  | 0.05 | 3.023 | 0.502 | 3 | 3.024 | 0.488 | 3 | 3.032 | 0.477 | 3 | 3.033 | 0.472 | 3 |
|  | 0.1 | 3.022 | 0.5 | 3 | 3.023 | 0.482 | 3 | 3.025 | 0.481 | 3 | 3.032 | 0.474 | 3 |
|  | 0.25 | 3.015 | 0.498 | 3 | 3.024 | 0.479 | 3 | 3.029 | 0.478 | 3 | 3.03 | 0.476 | 3 |
|  | 0.5 | 3.022 | 0.5 | 3 | 3.023 | 0.487 | 3 | 3.027 | 0.474 | 3 | 3.028 | 0.476 | 3 |
|  | 0.75 | 3.018 | 0.5 | 3 | 3.025 | 0.483 | 3 | 3.027 | 0.479 | 3 | 3.028 | 0.473 | 3 |
|  | 1 | 2.933 | 0.406 | 3 | 2.944 | 0.383 | 3 | 2.945 | 0.372 | 3 | 2.948 | 0.369 | 3 |
|  | 1.5 | 2.299 | 0.479 | 2 | 2.3 | 0.469 | 2 | 2.308 | 0.466 | 2 | 2.309 | 0.464 | 2 |
|  | 2 | 1.993 | 0.115 | 2 | 1.997 | 0.081 | 2 | 1.999 | 0.05 | 2 | 2.001 | 0.035 | 2 |
| 0.5 | 0 | 5.347 | 1.748 | 5 | 5.36 | 1.74 | 5 | 5.377 | 1.734 | 5 | 5.379 | 1.723 | 5 |
|  | 0.05 | 5.342 | 1.725 | 5 | 5.384 | 1.75 | 5 | 5.385 | 1.725 | 5 | 5.388 | 1.73 | 5 |
|  | 0.1 | 5.365 | 1.76 | 5 | 5.366 | 1.723 | 5 | 5.367 | 1.734 | 5 | 5.375 | 1.731 | 5 |
|  | 0.25 | 5.357 | 1.743 | 5 | 5.38 | 1.743 | 5 | 5.396 | 1.745 | 5 | 5.398 | 1.735 | 5 |
|  | 0.5 | 5.16 | 1.52 | 5 | 5.179 | 1.512 | 5 | 5.181 | 1.496 | 5 | 5.203 | 1.497 | 5 |
|  | 0.75 | 4.192 | 0.923 | 4 | 4.2 | 0.908 | 4 | 4.212 | 0.9 | 4 | 4.214 | 0.897 | 4 |
|  | 1 | 3.35 | 0.6 | 3 | 3.358 | 0.58 | 3 | 3.365 | 0.571 | 3 | 3.367 | 0.568 | 3 |
|  | 1.5 | 2.404 | 0.512 | 2 | 2.416 | 0.504 | 2 | 2.418 | 0.499 | 2 | 2.419 | 0.496 | 2 |
|  | 2 | 1.999 | 0.137 | 2 | 2.003 | 0.112 | 2 | 2.005 | 0.092 | 2 | 2.006 | 0.083 | 2 |
| 0.75 | 0 | 25.19 | 19.476 | 20 | 25.391 | 19.652 | 20 | 25.447 | 19.66 | 20 | 25.532 | 19.747 | 20 |
|  | 0.05 | 25.304 | 19.515 | 20 | 25.459 | 19.649 | 20 | 25.506 | 19.598 | 20 | 25.649 | 19.677 | 20 |
|  | 0.1 | 25.219 | 19.197 | 20 | 25.283 | 19.319 | 20 | 25.292 | 19.728 | 20 | 25.371 | 19.172 | 20 |
|  | 0.25 | 20.538 | 14.731 | 17 | 20.646 | 14.677 | 17 | 20.814 | 14.96 | 17 | 20.939 | 14.786 | 17 |
|  | 0.5 | 8.242 | 3.836 | 8 | 8.302 | 3.895 | 8 | 8.318 | 3.863 | 8 | 8.319 | 3.864 | 8 |
|  | 0.75 | 4.685 | 1.489 | 5 | 4.689 | 1.485 | 4 | 4.703 | 1.464 | 5 | 4.775 | 1.461 | 4 |
|  | 1 | 3.461 | 0.852 | 3 | 3.467 | 0.84 | 3 | 3.476 | 0.836 | 3 | 3.487 | 0.836 | 3 |
|  | 1.5 | 2.454 | 0.55 | 2 | 2.461 | 0.544 | 2 | 2.462 | 0.538 | 2 | 2.469 | 0.54 | 2 |
|  | 2 | 2.039 | 0.24 | 2 | 2.041 | 0.221 | 2 | 2.044 | 0.215 | 2 | 2.045 | 0.211 | 2 |
| 0.85 | 0 | 90.83 | 85.047 | 64 | 90.851 | 86.013 | 65 | 90.868 | 84.391 | 65 | 91.007 | 85.833 | 66 |
|  | 0.05 | 88.786 | 84.427 | 64 | 89.007 | 83.233 | 64 | 89.974 | 84.313 | 64 | 90.056 | 83.811 | 64 |
|  | 0.1 | 81.515 | 76.325 | 59 | 82.031 | 75.537 | 59 | 82.225 | 77.032 | 59 | 82.233 | 76.275 | 59 |
|  | 0.25 | 34.301 | 28.27 | 26 | 34.318 | 28.167 | 26 | 34.508 | 28.564 | 26 | 34.661 | 28.359 | 26 |
|  | 0.5 | 8.71 | 4.56 | 8 | 8.729 | 4.592 | 8 | 8.74 | 4.551 | 8 | 8.743 | 4.524 | 8 |
|  | 0.75 | 4.748 | 1.687 | 5 | 4.751 | 1.667 | 5 | 4.783 | 1.685 | 5 | 4.784 | 1.691 | 5 |
|  | 1 | 3.487 | 0.957 | 3 | 3.497 | 0.949 | 3 | 3.51 | 0.95 | 3 | 3.52 | 0.939 | 3 |
|  | 1.5 | 2.481 | 0.581 | 2 | 2.483 | 0.569 | 2 | 2.485 | 0.563 | 2 | 2.489 | 0.564 | 2 |
|  | 2 | 2.062 | 0.28 | 2 | 2.063 | 0.263 | 2 | 2.066 | 0.255 | 2 | 2.068 | 0.255 | 2 |
| 0.9 | 0 | 195.338 | 191.644 | 136 | 195.928 | 189.794 | 136 | 196.633 | 193.194 | 138 | 196.849 | 193.19 | 137 |
|  | 0.05 | 179.18 | 174.589 | 125 | 179.34 | 175.235 | 126 | 180.032 | 177.019 | 125 | 180.211 | 176.458 | 126 |
|  | 0.1 | 141.915 | 137.709 | 99 | 141.921 | 138.876 | 100 | 141.943 | 137.486 | 100 | 142.068 | 135.819 | 100 |
|  | 0.25 | 37.032 | 31.65 | 28 | 37.15 | 31.685 | 28 | 37.817 | 32.364 | 28 | 37.999 | 31.744 | 28 |
|  | 0.5 | 8.746 | 4.79 | 8 | 8.804 | 4.814 | 8 | 8.814 | 4.728 | 8 | 8.821 | 4.745 | 8 |
|  | 0.75 | 4.788 | 1.798 | 5 | 4.791 | 1.787 | 5 | 4.806 | 1.77 | 5 | 4.832 | 1.782 | 5 |
|  | 1 | 3.505 | 1.013 | 3 | 3.511 | 0.996 | 3 | 3.519 | 1.006 | 3 | 3.53 | 0.997 | 3 |
|  | 1.5 | 2.486 | 0.595 | 2 | 2.488 | 0.584 | 2 | 2.498 | 0.584 | 2 | 2.508 | 0.581 | 2 |
|  | 2 | 2.075 | 0.3 | 2 | 2.077 | 0.287 | 2 | 2.08 | 0.28 | 2 | 2.087 | 0.272 | 2 |
| 1 | 0 | 370.042 | 369.612 | 259 | 370.073 | 372.255 | 256 | 370.123 | 370.394 | 257 | 370.166 | 368.623 | 258 |
|  | 0.05 | 295.481 | 294.392 | 205 | 295.577 | 295.612 | 200 | 295.697 | 293.994 | 206 | 295.809 | 294.529 | 206 |
|  | 0.1 | 170.789 | 168.366 | 118 | 171.473 | 167.739 | 120 | 172.134 | 167.744 | 120 | 172.907 | 168.05 | 122 |
|  | 0.25 | 34.249 | 29.505 | 25 | 34.775 | 29.991 | 26 | 34.856 | 29.757 | 26 | 35.439 | 29.507 | 26 |
|  | 0.5 | 8.776 | 5.056 | 8 | 8.777 | 5.078 | 8 | 8.801 | 5.115 | 8 | 8.817 | 5.06 | 8 |
|  | 0.75 | 4.816 | 1.961 | 4 | 4.828 | 1.972 | 5 | 4.832 | 1.947 | 5 | 4.856 | 1.976 | 4 |
|  | 1 | 3.547 | 1.134 | 3 | 3.552 | 1.119 | 3 | 3.555 | 1.112 | 3 | 3.558 | 1.105 | 3 |
|  | 1.5 | 2.51 | 0.629 | 2 | 2.515 | 0.625 | 2 | 2.517 | 0.616 | 2 | 2.525 | 0.615 | 2 |
|  | 2 | 2.096 | 0.33 | 2 | 2.099 | 0.318 | 2 | 2.1 | 0.314 | 2 | 2.101 | 0.307 | 2 |
| 1.05 | 0 | 198.82 | 197.585 | 139 | 198.898 | 197.002 | 139 | 198.901 | 198.718 | 138 | 199.299 | 199.012 | 138 |
|  | 0.05 | 171.144 | 170.862 | 118 | 172.54 | 170.088 | 121 | 173.2 | 172.906 | 120 | 173.36 | 173.083 | 120 |
|  | 0.1 | 116.854 | 115.864 | 82 | 117.12 | 114.416 | 83 | 117.15 | 113.348 | 82 | 117.25 | 113.446 | 82 |
|  | 0.25 | 30.697 | 26.546 | 23 | 30.715 | 26.576 | 23 | 30.806 | 26.438 | 23 | 30.836 | 26.559 | 23 |
|  | 0.5 | 8.631 | 5.144 | 7 | 8.688 | 5.103 | 8 | 8.71 | 5.138 | 8 | 8.714 | 5.152 | 8 |
|  | 0.75 | 4.855 | 2.072 | 4 | 4.865 | 2.073 | 4 | 4.873 | 2.052 | 4 | 4.886 | 2.045 | 5 |
|  | 1 | 3.553 | 1.178 | 3 | 3.57 | 1.171 | 3 | 3.572 | 1.166 | 3 | 3.583 | 1.152 | 3 |
|  | 1.5 | 2.52 | 0.645 | 2 | 2.525 | 0.633 | 2 | 2.528 | 0.629 | 2 | 2.529 | 0.632 | 2 |
|  | 2 | 2.11 | 0.348 | 2 | 2.113 | 0.34 | 2 | 2.115 | 0.331 | 2 | 2.118 | 0.332 | 2 |
| 1.1 | 0 | 94.332 | 91.902 | 66 | 94.456 | 93.09 | 66 | 94.475 | 91.846 | 67 | 94.807 | 91.964 | 67 |
|  | 0.05 | 85.999 | 83.7 | 60 | 87.191 | 84.515 | 62 | 87.355 | 84.291 | 62 | 87.594 | 83.947 | 62 |
|  | 0.1 | 68.322 | 65.886 | 49 | 68.358 | 65.586 | 49 | 68.411 | 65.347 | 48 | 68.869 | 66.069 | 49 |
|  | 0.25 | 26.154 | 22.14 | 20 | 26.256 | 22.248 | 20 | 26.317 | 22.177 | 20 | 26.346 | 22.39 | 20 |
|  | 0.5 | 8.447 | 5.2 | 7 | 8.5 | 5.145 | 7 | 8.553 | 5.202 | 7 | 8.577 | 5.139 | 7 |
|  | 0.75 | 4.84 | 2.144 | 4 | 4.859 | 2.105 | 4 | 4.88 | 2.117 | 4 | 4.892 | 2.119 | 4 |
|  | 1 | 3.554 | 1.224 | 3 | 3.569 | 1.214 | 3 | 3.581 | 1.218 | 3 | 3.588 | 1.203 | 3 |
|  | 1.5 | 2.536 | 0.666 | 2 | 2.541 | 0.657 | 2 | 2.542 | 0.654 | 2 | 2.544 | 0.652 | 2 |
|  | 2 | 2.121 | 0.362 | 2 | 2.124 | 0.356 | 2 | 2.125 | 0.344 | 2 | 2.129 | 0.346 | 2 |
| 1.15 | 0 | 49.08 | 45.469 | 36 | 49.189 | 46.621 | 36 | 49.274 | 45.941 | 35 | 49.716 | 46.466 | 35 |
|  | 0.05 | 47.07 | 43.859 | 34 | 47.267 | 43.758 | 34 | 47.375 | 44.026 | 34 | 47.465 | 43.573 | 34 |
|  | 0.1 | 41.229 | 38.231 | 30 | 41.358 | 37.707 | 30 | 41.401 | 38.218 | 30 | 41.471 | 38.134 | 30 |
|  | 0.25 | 21.36 | 17.542 | 16 | 21.41 | 17.786 | 17 | 21.482 | 17.52 | 17 | 21.546 | 17.684 | 17 |
|  | 0.5 | 8.264 | 5.048 | 7 | 8.285 | 5.063 | 7 | 8.31 | 5.034 | 7 | 8.403 | 5.049 | 7 |
|  | 0.75 | 4.857 | 2.202 | 4 | 4.862 | 2.188 | 4 | 4.878 | 2.195 | 4 | 4.955 | 2.175 | 4 |
|  | 1 | 3.565 | 1.256 | 3 | 3.577 | 1.263 | 3 | 3.579 | 1.261 | 3 | 3.59 | 1.249 | 3 |
|  | 1.5 | 2.542 | 0.682 | 2 | 2.544 | 0.671 | 2 | 2.553 | 0.672 | 2 | 2.555 | 0.668 | 2 |
|  | 2 | 2.131 | 0.376 | 2 | 2.132 | 0.363 | 2 | 2.139 | 0.363 | 2 | 2.148 | 0.36 | 2 |
| 1.25 | 0 | 19.71 | 16.444 | 15 | 19.74 | 16.332 | 15 | 19.752 | 16.219 | 15 | 19.842 | 16.406 | 15 |
|  | 0.05 | 19.343 | 15.887 | 15 | 19.359 | 15.874 | 15 | 19.568 | 16.128 | 15 | 19.605 | 16.296 | 15 |
|  | 0.1 | 18.503 | 15.163 | 14 | 18.515 | 15.226 | 14 | 18.583 | 15.314 | 14 | 18.683 | 15.351 | 14 |
|  | 0.25 | 13.781 | 10.423 | 11 | 13.924 | 10.527 | 11 | 13.942 | 10.586 | 11 | 13.989 | 10.342 | 11 |
|  | 0.5 | 7.414 | 4.506 | 6 | 7.444 | 4.518 | 6 | 7.478 | 4.528 | 6 | 7.488 | 4.537 | 6 |
|  | 0.75 | 4.757 | 2.25 | 4 | 4.763 | 2.242 | 4 | 4.767 | 2.22 | 4 | 4.78 | 2.197 | 4 |
|  | 1 | 3.574 | 1.355 | 3 | 3.575 | 1.334 | 3 | 3.577 | 1.324 | 3 | 3.579 | 1.342 | 3 |
|  | 1.5 | 2.562 | 0.717 | 2 | 2.566 | 0.705 | 2 | 2.577 | 0.712 | 2 | 2.579 | 0.696 | 2 |
|  | 2 | 2.154 | 0.405 | 2 | 2.157 | 0.398 | 2 | 2.158 | 0.387 | 2 | 2.159 | 0.389 | 2 |
| 1.5 | 0 | 6.759 | 4.232 | 6 | 6.762 | 4.252 | 6 | 6.817 | 4.247 | 6 | 6.834 | 4.322 | 6 |
|  | 0.05 | 6.605 | 4.272 | 6 | 6.77 | 4.204 | 6 | 6.78 | 4.196 | 6 | 6.8 | 4.245 | 6 |
|  | 0.1 | 6.515 | 4.193 | 6 | 6.652 | 4.116 | 6 | 6.722 | 4.216 | 6 | 6.869 | 4.138 | 6 |
|  | 0.25 | 6.297 | 3.776 | 5 | 6.299 | 3.83 | 5 | 6.329 | 3.74 | 5 | 6.332 | 3.84 | 5 |
|  | 0.5 | 5.175 | 2.846 | 5 | 5.176 | 2.83 | 5 | 5.179 | 2.815 | 5 | 5.21 | 2.847 | 5 |
|  | 0.75 | 4.141 | 1.973 | 4 | 4.159 | 1.961 | 4 | 4.169 | 1.949 | 4 | 4.188 | 1.953 | 4 |
|  | 1 | 3.307 | 1.39 | 3 | 3.396 | 1.361 | 3 | 3.414 | 1.374 | 3 | 3.421 | 1.361 | 3 |
|  | 1.5 | 2.58 | 0.779 | 2 | 2.582 | 0.767 | 2 | 2.589 | 0.769 | 2 | 2.695 | 0.775 | 2 |
|  | 2 | 2.194 | 0.465 | 2 | 2.199 | 0.459 | 2 | 2.204 | 0.454 | 2 | 2.302 | 0.451 | 2 |
| 2 | 0 | 2.125 | 1.976 | 2 | 3.273 | 1.541 | 3 | 3.296 | 1.498 | 3 | 3.377 | 1.525 | 3 |
|  | 0.05 | 2.824 | 1.833 | 3 | 3.048 | 1.538 | 3 | 3.268 | 1.489 | 3 | 3.274 | 1.448 | 3 |
|  | 0.1 | 2.392 | 1.955 | 2 | 3.189 | 1.628 | 3 | 3.246 | 1.493 | 3 | 3.365 | 1.503 | 3 |
|  | 0.25 | 2.949 | 1.671 | 3 | 3.073 | 1.641 | 3 | 3.216 | 1.397 | 3 | 3.303 | 1.619 | 3 |
|  | 0.5 | 2.777 | 1.561 | 3 | 2.988 | 1.411 | 3 | 3.142 | 1.345 | 3 | 3.225 | 1.325 | 3 |
|  | 0.75 | 2.307 | 1.597 | 2 | 2.865 | 1.324 | 3 | 2.899 | 1.235 | 3 | 2.914 | 1.409 | 2 |
|  | 1 | 2.184 | 1.468 | 2 | 2.796 | 1.124 | 3 | 2.817 | 1.134 | 3 | 2.986 | 1.013 | 2 |
|  | 1.5 | 2.351 | 0.87 | 2 | 2.41 | 0.777 | 2 | 2.431 | 0.801 | 2 | 2.447 | 0.774 | 2 |
|  | 2 | 1.467 | 1.136 | 2 | 2.123 | 0.65 | 2 | 2.178 | 0.615 | 2 | 2.186 | 0.512 | 2 |
| 2.5 | 0 | 0.273 | 0.898 | 0 | 1.28 | 1.415 | 2 | 1.429 | 1.548 | 2 | 1.944 | 1.264 | 2 |
|  | 0.05 | 1.272 | 1.444 | 0 | 1.62 | 1.48 | 2 | 2 | 1.192 | 2 | 2.368 | 1.422 | 2 |
|  | 0.1 | 1.294 | 1.503 | 0 | 1.853 | 1.329 | 2 | 2.42 | 1.179 | 2 | 2.494 | 1.163 | 2 |
|  | 0.25 | 0.664 | 1.22 | 0 | 1.597 | 1.382 | 2 | 1.61 | 1.498 | 2 | 1.643 | 1.393 | 2 |
|  | 0.5 | 0.782 | 1.311 | 0 | 1.351 | 1.353 | 2 | 1.75 | 1.512 | 2 | 1.941 | 1.103 | 2 |
|  | 0.75 | 1.241 | 1.403 | 0 | 1.487 | 1.442 | 2 | 1.955 | 1.24 | 2 | 2.096 | 1.082 | 2 |
|  | 1 | 1.254 | 1.332 | 2 | 1.373 | 1.455 | 2 | 1.883 | 1.267 | 2 | 1.952 | 1.496 | 2 |
|  | 1.5 | 1.332 | 1.19 | 2 | 1.408 | 1.045 | 2 | 1.974 | 1.046 | 2 | 2.029 | 1.035 | 2 |
|  | 2 | 0.105 | 0.437 | 0 | 1.462 | 0.955 | 2 | 1.591 | 1.095 | 2 | 1.802 | 0.809 | 2 |
| 3 | 0 | 0.115 | 0.527 | 0 | 1.054 | 1.189 | 0 | 1.283 | 1.205 | 2 | 1.291 | 1.054 | 2 |
|  | 0.05 | 0.077 | 0.386 | 0 | 0.547 | 1.022 | 0 | 1.34 | 1.203 | 2 | 1.568 | 1.015 | 2 |
|  | 0.1 | 0.011 | 0.794 | 0 | 0.06 | 0.424 | 0 | 1.136 | 1.091 | 2 | 1.348 | 1.265 | 2 |
|  | 0.25 | 0.02 | 0.2 | 0 | 0.672 | 1.186 | 0 | 1.2 | 1.203 | 2 | 1.22 | 1.152 | 2 |
|  | 0.5 | 0.028 | 0.782 | 0 | 0.04 | 0.283 | 0 | 1.128 | 1.151 | 2 | 1.143 | 1.153 | 2 |
|  | 0.75 | 0.069 | 0.404 | 0 | 0.298 | 0.755 | 0 | 1.356 | 1.246 | 2 | 1.41 | 1.021 | 2 |
|  | 1 | 0.03 | 0.729 | 0 | 0.039 | 0.28 | 0 | 1.15 | 1.189 | 2 | 1.182 | 1.14 | 2 |
|  | 1.5 | 0.03 | 0.299 | 0 | 0.508 | 0.954 | 0 | 1.105 | 1.181 | 1 | 1.227 | 1.193 | 2 |
|  | 2 | 0.036 | 0.359 | 0 | 0.058 | 0.308 | 0 | 1.362 | 1.055 | 2 | 1.53 | 1.113 | 2 |


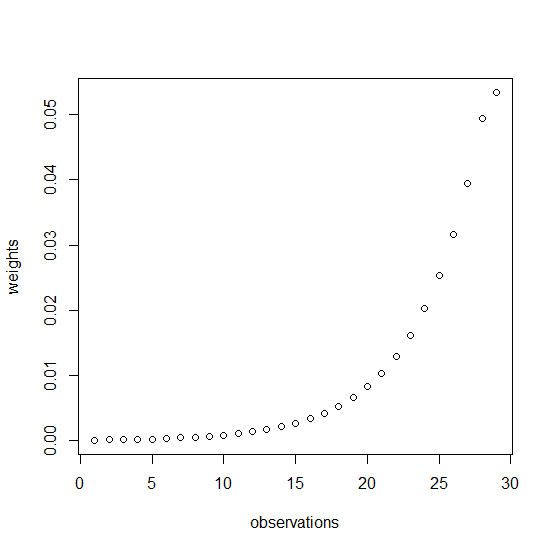


Supplementary Figure A: Weighting pattern of EWMA statistic with weighted moving averages for $\lambda=0.2$

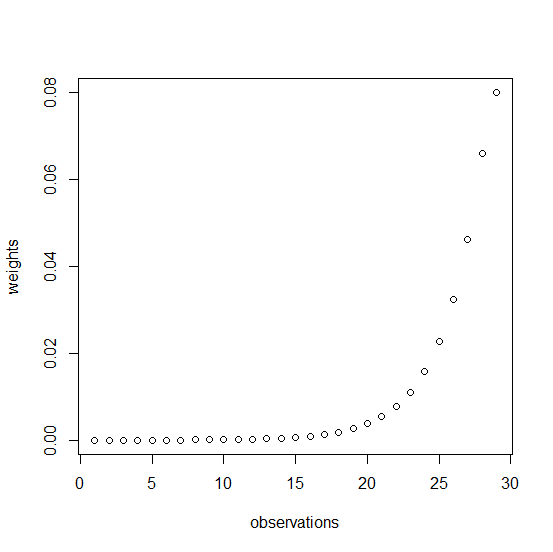


Supplementary Figure B: Weighting pattern of EWMA statistic with weighted moving averages for $\lambda=0.3$
